# Neural ablation of the *PARK10* candidate *Plpp3* leads to dopaminergic transmission deficits without neurodegeneration

**DOI:** 10.1038/srep24028

**Published:** 2016-04-11

**Authors:** Sandra Gómez-López, Ana Valeria Martínez-Silva, Teresa Montiel, Daniel Osorio-Gómez, Federico Bermúdez-Rattoni, Lourdes Massieu, Diana Escalante-Alcalde

**Affiliations:** 1 Instituto de Fisiología Celular, División de Neurociencias, Universidad Nacional Autónoma de México, Circuito Exterior s/n, Ciudad Universitaria, Mexico City, 04510, Mexico

## Abstract

Parkinson’s disease (PD) is a multifactorial neurodegenerative disorder, characterised by the progressive loss of midbrain dopaminergic neurons and a variety of motor symptoms. The gene coding for the phospholipid phosphatase 3, *PLPP3* (formerly *PPAP2B* or *LPP3*), maps within the *PARK10* locus, a region that has been linked with increased risk to late-onset PD. PLPP3 modulates the levels of a range of bioactive lipids controlling fundamental cellular processes within the central nervous system. Here we show that PLPP3 is enriched in astroglial cells of the adult murine ventral midbrain. Conditional inactivation of *Plpp3* using a *Nestin::Cre* driver results in reduced mesencephalic levels of sphingosine-1-phosphate receptor 1 (S1P_1_), a well-known mediator of pro-survival responses. Yet, adult PLPP3-deficient mice exhibited no alterations in the number of dopaminergic neurons or in the basal levels of striatal extracellular dopamine (DA). Potassium-evoked DA overflow in the striatum, however, was significantly decreased in mutant mice. Locomotor evaluation revealed that, although PLPP3-deficient mice exhibit motor impairment, this is not progressive or responsive to acute L-DOPA therapy. These findings suggest that disruption of *Plpp3* during early neural development leads to dopaminergic transmission deficits in the absence of nigrostriatal degeneration, and without causing an age-related locomotor decline consistent with PD.

Parkinson’s disease (PD) is a neurodegenerative disorder clinically characterised by slowness of movement, muscle rigidity, resting tremor and postural instability. Hallmark features of PD include progressive loss of ventral midbrain dopaminergic neurons and appearance of insoluble protein inclusions known as Lewy bodies in surviving neurons. Yet, the pathogenesis of PD is not fully understood and treatment options are still limited to symptomatic therapy, such as L-DOPA^1^.

Linkage and genome-wide association analyses have identified several PD-associated loci. However, in many cases the identity of the specific PD risk gene remains unclear^2^. Indeed, a heritability study estimated that up to 40% of the variation in PD susceptibility is due to as-yet unidentified genes^3^. The *PARK10* locus has been implicated in the susceptibility to late-onset PD^4^,^5^,^6^,^7^,^8^. However, the identity of the gene(s) conferring such susceptibility has yet to be defined. Among the genes that map within this region, and that have been found to be dysregulated in patients with PD^9^, is *PLPP3*, which encodes the phospholipid phosphatase 3. PLPP3 is an integral membrane glycoprotein that hydrolyses and thereby modulates the availability of several extracellular lipid phosphates, including lysophosphatidic acid (LPA) and sphingosine-1-phosphate (S1P)^10^. Extracellular LPA and S1P signal through G protein-coupled receptors (GPCRs) and activate multiple signalling pathways controlling proliferation, survival, migration and differentiation along the neuronal and glial cell lineages^11^,^12^,^13^.

S1P is a well-established pro-survival molecule in mammalian cells. It is synthesised intracellularly via sphingosine kinase-mediated phosphorylation of sphingosine, and signals through five different receptors (S1P_1–5_)^14^. Gene expression profiling of dopaminergic neurons isolated from the substantia nigra (SN) of patients with PD and control subjects revealed downregulation of sphingosine kinase 2 (*SPHK2*) transcripts in PD samples^15^. Accordingly, levels of SPHK2, S1P and S1P_1_ were reduced in the SN of a 1-methyl-4-phenyl-1,2,3,6-tetrahydropyridine (MPTP)-induced PD murine model^16^, and addition of exogenous S1P to 1-methyl-4-phenylpyridinium (MPP^+^)-treated dopaminergic neuron cultures was found to exert a neuroprotective effect^16^,^17^,^18^. These observations suggest that dysregulation of S1P metabolism could play a role in PD pathogenesis. Since PLPP3 modulates S1P metabolism/signalling in the brain^19^, it is conceivable that PLPP3 might be implicated in PD.

We previously showed that mice lacking PLPP3 in the neural lineage exhibit impaired motor performance from one month of age. Abnormalities in cerebellar foliation in mutant animals suggested that structural or electrophysiological alterations of the cerebellum, the brain structure that regulates skilled movement, may be causal to these defects^19^. Here we investigated whether the motor deficit of PLPP3-deficient mice may be related to parkinsonism. We found that, although PLPP3 is expressed in the ventral midbrain, *Nestin::Cre* mediated inactivation of *Plpp3* does not substantially affect the development and maintenance of the nigrostriatal pathway. Additionally, we show that the motor phenotype of PLPP3-deficient mice is not progressive or responsive to acute L-DOPA therapy. However, potassium-evoked dopamine (DA) overflow in the striatum is markedly reduced in mice lacking PLPP3. Collectively, these data suggest that disruption of *Plpp3* during embryonic central nervous system (CNS) development does not cause ventral midbrain DA neuron loss or locomotor impairments consistent with PD, but results in dopaminergic transmission deficits that might contribute to the pathology under specific contexts.

## Results

### *Plpp3* is expressed in the ventral midbrain

**Figure 1:**
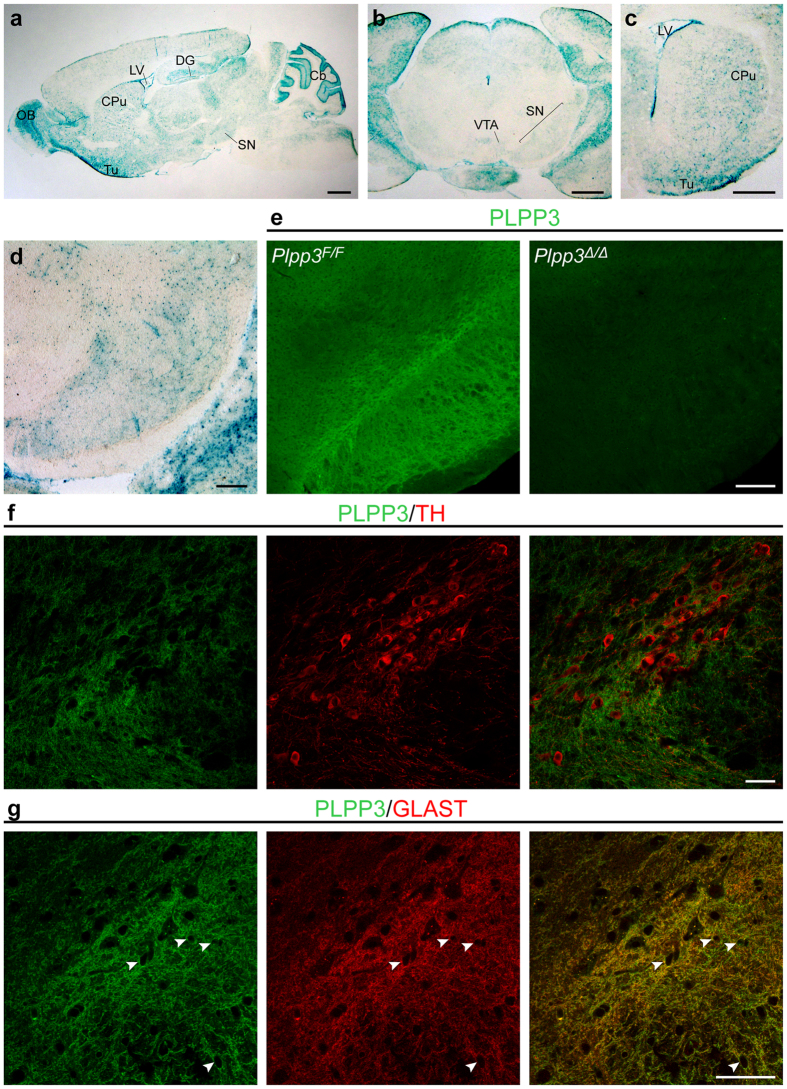
*Plpp3*/PLPP3 expression in the murine ventral midbrain. **(a–d)** X-gal staining on 4-week-old *Plpp3*^+*/lacZ*^ mouse brain sections. Scale bars: (**a–c**) 800 μm; (**d**) 200 μm. **(e)** PLPP3 immunostaining in the adult ventral midbrain of a control mouse (*Plpp3*^*F/F*^) and a littermate lacking *Plpp3* in the neural lineage (*Plpp3*^*F/F*^; *Nestin::Cre* or *Plpp3*^Δ*/*Δ^). Note lack of PLPP3 immunoreactivity in the mutant brain. Scale bar, 200 μm. Double immunofluorescence for **(f)** PLPP3 and TH or **(g)** PLPP3 and GLAST in the 16-week-old murine ventral midbrain. Arrowheads show evident overlap of both markers. Scale bars, 50 μm. Cb, cerebellum; CPu, caudate putamen; DG, dentate gyrus; LV, lateral ventricle; OB, olfactory bulb; SN, substantia nigra; Tu, olfactory tubercle; VTA, ventral tegmental area.

*PARK10* spans up to a 9.5 megabase region on chromosome 1p32 (50700000-62300000). Alterations in one or more genes within this locus may confer susceptibility to late-onset PD^4^,^5^. To investigate whether *PLPP3* (NC_000001.11: 56494747–56579584) may be implicated in PD pathogenesis, we analysed its expression in the mouse midbrain. Using a *Plpp3*^*lacZ*^ reporter allele and immunostaining, we previously showed that both β-galactosidase reporter activity and PLPP3 protein are detected in the developing midbrain between embryonic days (E) 10.5 and 12.5^20^, when the great majority of DA neurons of the SN arise^21^. X-gal staining on brain sections from juvenile (4-week-old) and adult (12-week-old) *Plpp3*^*lacZ*^ mice revealed moderate but well-defined β-galactosidase expression along the nigrostriatal pathway, from the SN to the caudate putamen (CPu) (Fig. 1a–d). Additionally, strong reporter expression was seen in the cerebellum, olfactory bulb and ventricular system, as previously reported^19^, as well as in the hippocampal dentate gyrus and olfactory tubercle (Fig. 1a,c). Immunostaining for PLPP3, using a previously characterised antibody^19^,^22^, showed clear staining in the adult SN (Fig. 1e and Fig. S1), demonstrating that during adulthood, protein distribution in the ventral midbrain correlates with reporter activity.

Transcriptional profiling analyses of astroglial, neuronal and oligodendroglial cell populations acutely purified from the postnatal and adult mouse forebrain indicate that *Plpp3* transcripts are significantly enriched in astrocytes in comparison to neurons and oligodendrocytes^23^,^24^. In the cerebellum, expression of PLPP3 protein is restricted to a specialised type of GLAST^+^ and S100β^+^ glial cell, the Bergmann glia, and is absent from Purkinje cells, interneurons and granule cells^19^. To determine the identity of the cells expressing PLPP3 in the adult ventral midbrain, we performed immunofluorescence analyses for PLPP3 in combination with antibodies detecting either tyrosine hydroxylase (TH), the rate-limiting enzyme in the synthesis of dopamine, or the glutamate-aspartate transporter GLAST, which is enriched at the plasma membrane of astrocytes. We found that in the SN and ventral tegmental area (VTA), PLPP3 shows a similar expression pattern to that of GLAST, and while being in close contact with TH^+^ neurons, no obvious immunoreactivity was detected in these cells (Fig. 1f,g). This suggests that PLPP3 might modulate paracrine signals between astroglia and neurons by regulating the extracellular availability of bioactive lipid phosphates, such as S1P.

### Development of midbrain DA neurons in the absence of PLPP3

**Figure 2:**
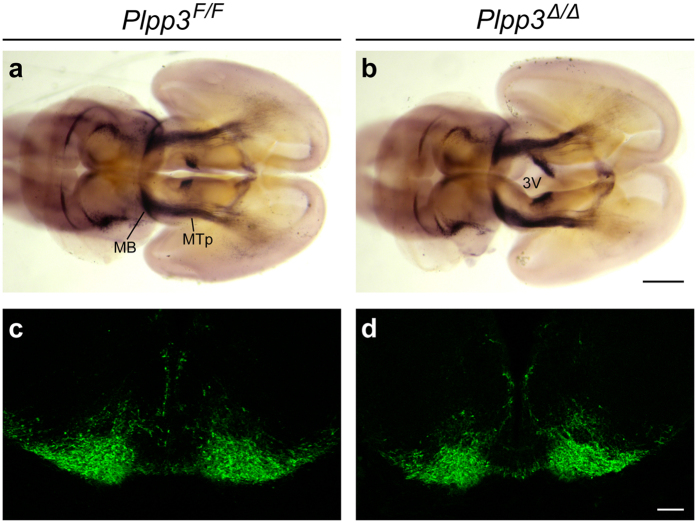
TH immunohistochemistry in the PLPP3-deficient developing mesencephalon. Antibody staining on **(a**,**b)** whole-mount preparations (ventral view) and **(c**,**d)** coronal sections of E14.5 brains showing comparable TH immunoreactivity in the *Plpp3*^Δ*/*Δ^ and control midbrain. Scale bars: (**a**,**b**) 500 μm; (**c**,**d**) 100 μm. MB, midbrain; MTp, mesotelencephalic projections; 3V, third ventricle.

To investigate whether PLPP3 is required for the development of midbrain DA neurons, we used a mouse line bearing floxed *Plpp3* alleles (*Plpp3*^*F/F*^)^25^ to conditionally deplete *Plpp3* in CNS progenitor cells. Since *Plpp3* is expressed in the prospective midbrain^20^, its ablation at early embryonic stages could potentially affect the proper specification of this structure. Therefore, we inactivated *Plpp3* using a *Nestin::Cre* transgenic mouse line^26^ that exhibits Cre recombinase activity in the ventral midbrain from E10.5^27^. Efficient excision of *Plpp3* in the CNS of *Plpp3*^*F/F*^; *Nestin::Cre* mice (hereafter referred to as *Plpp3*^Δ*/*Δ^) has been previously demonstrated^19^ and was confirmed by PCR of genomic DNA, western blot and immunostaining (Fig. 1e, Figs S1 and S2). Expression of TH in E14.5 *Plpp3*^Δ*/*Δ^ and control *Plpp3*^*F/F*^ mouse brains was analysed by immunostaining of whole-mount preparations and tissue sections across the mesencephalon. There were no substantial differences throughout the TH^+^ domains between genotypes (Fig. 2). However, we found that in the PLPP3-deficient brains the third ventricle was enlarged when compared with non-excised controls (Fig. 2b).

### Lack of PLPP3 in the adult nigrostriatal pathway alters S1P/S1P_1_ signalling without affecting DA neuron survival

**Figure 3:**
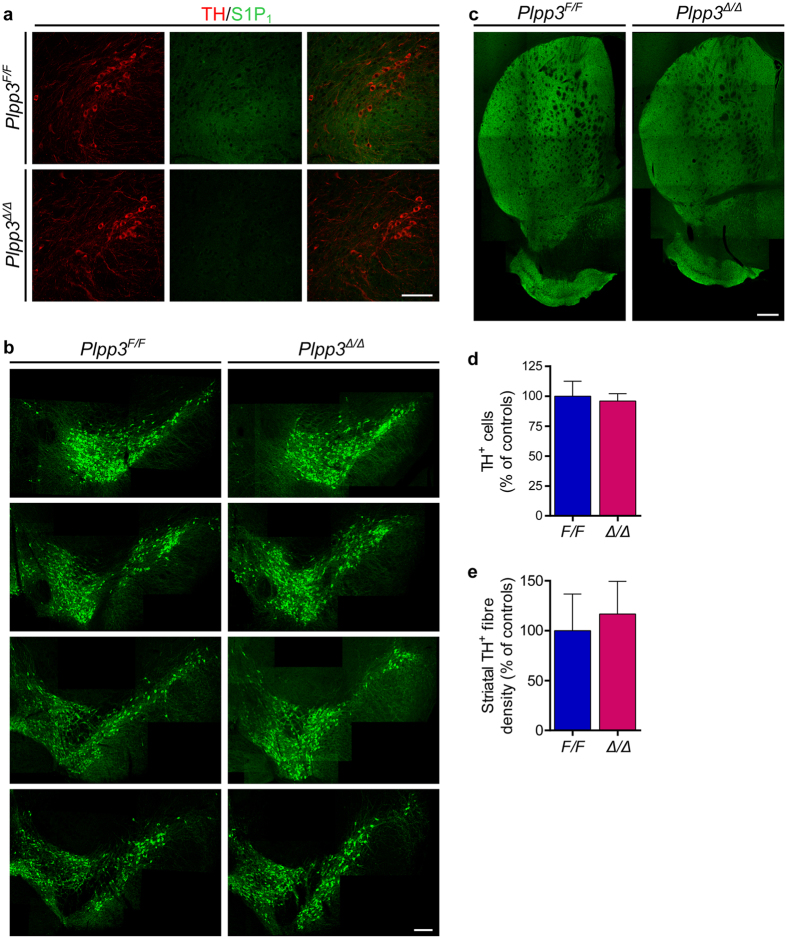
PLPP3-deficiency in the adult ventral midbrain. **(a)** Immunostaining for S1P_1_ and TH on coronal brain sections from 16-week-old mice shows down-regulation of S1P_1_ in the *Plpp3*^Δ*/*Δ^ ventral midbrain in comparison to *Plpp3*^*F/F*^ control. Scale bar, 100 μm. TH immunofluorescence in the **(b)** ventral midbrain and **(c)** striatum of *Plpp3*^*F/F*^ and *Plpp3*^Δ*/*Δ^ mice. Scale bars: SN-VTA, 200 μm; striatum, 400 μm. **(d)** Stereological quantitation of TH^+^ cell bodies in the SN and VTA of *Plpp3*^*F/F*^ and *Plpp3*^Δ*/*Δ^ mice at 16 weeks of age. *Plpp3*^*F/F*^ *n* = 3, *Plpp3*^Δ*/*Δ^ *n* = 4. **(e)** Assessment of TH^+^ fibre density in the dorsolateral striatum of *Plpp3*^*F/F*^ and *Plpp3*^Δ*/*Δ^ mice at the same age. *Plpp3*^*F/F*^ *n* = 4, *Plpp3*^Δ*/*Δ^ *n* = 5. Values represent mean ± standard deviation (SD). There are no statistically significant differences between genotypes in either (**d**) *p* = 0.5841 or (**e**) *p* = 0.4931 (*t* tests).

S1P/S1P_1_ signalling has been shown to exert a neuroprotective effect on DA neurons^16^,^18^ and aberrant sphingolipid metabolism has been implicated in distinct neurodegenerative disorders^28^,^29^. Moreover, *S1PR1* was recently proposed as a candidate gene for a newly identified PD susceptibility locus^30^, suggesting a potential role for S1P_1_ signalling in regulating midbrain DA neuron survival and/or function. We previously showed that depletion of PLPP3 in the neural lineage leads to downregulation of S1P_1_ in the cerebellum^19^. Immunofluorescence analysis of brain sections from 16-week-old *Plpp3*^Δ*/*Δ^ and control mice revealed that neural deficiency of PLPP3 also causes a pronounced decrease of S1P_1_ levels in the ventral midbrain (Fig. 3a and Fig. S1). To determine if the absence of PLPP3 and the concomitant reduction of mesencephalic S1P_1_ levels affect the integrity of the adult nigrostriatal pathway, we performed TH immunostaining. We found no obvious differences in the distribution of ventral midbrain DA neurons between *Plpp3*^Δ*/*Δ^ and non-excised animals (Fig. 3b). Stereological quantitation of TH^+^ cell bodies in the SN and VTA showed comparable numbers of DA neurons between genotypes (Fig. 3d). Moreover, densitometric analyses of TH^+^ innervation in the striatum revealed no significant differences among genotypes (Fig. 3c,e). Thus, despite displaying a motor deficit from one month of age^19^, the integrity of the nigrostriatal pathway in *Plpp3*^Δ*/*Δ^ mice seems unaffected at least up to 16 weeks of age.

**Figure 4:**
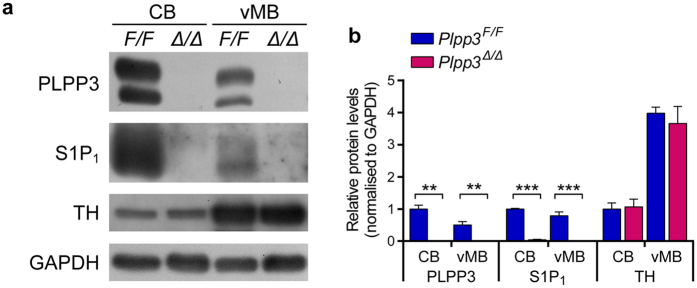
Dysregulation of S1P_1_ in the adult PLPP3-deficient midbrain. **(a**,**b)** Western blot analyses of ventral midbrain (vMB) tissue from 12-month-old mice show loss of PLPP3 and reduced levels of S1P_1_ in samples from *Plpp3*^Δ*/*Δ^ animals when compared with those from *Plpp3*^*F/F*^ controls. Note the presence of both the glycosylated (upper band) and not glycosylated (lower band) forms of PLPP3. Protein extracts obtained from the cerebellum (CB) are shown as controls. Plots represent mean of three independent experiments ± SD. CB *Plpp3*^*F/F*^ = 1. **p < 0.005, ***p < 0.0005 (*t* tests).

Since loss of midbrain DA neurons in patients with PD occurs progressively, we measured expression levels of TH in the ventral midbrain of 10- to 12-month-old *Plpp3*^Δ*/*Δ^ and *Plpp3*^*F/F*^ mice. Consistent with our observations in 16-week-old PLPP3-deficient mice, western blot analyses showed that at 10- to 12-months of age, *Plpp3*^Δ*/*Δ^ mice exhibit a considerable reduction of S1P_1_ levels in the ventral midbrain, but no differences in TH expression when compared to age-matched controls (Fig. 4). These data suggest that, despite leading to S1P_1_ dysregulation in the ventral midbrain, PLPP3 deficiency does not cause a substantial loss of TH^+^ cells in this region.

### Unchanged levels of astrocyte reactivity and Fluoro-Jade^+^ cells in the SN of adult *Plpp3* ^Δ*/*Δ^ mice

**Figure 5:**
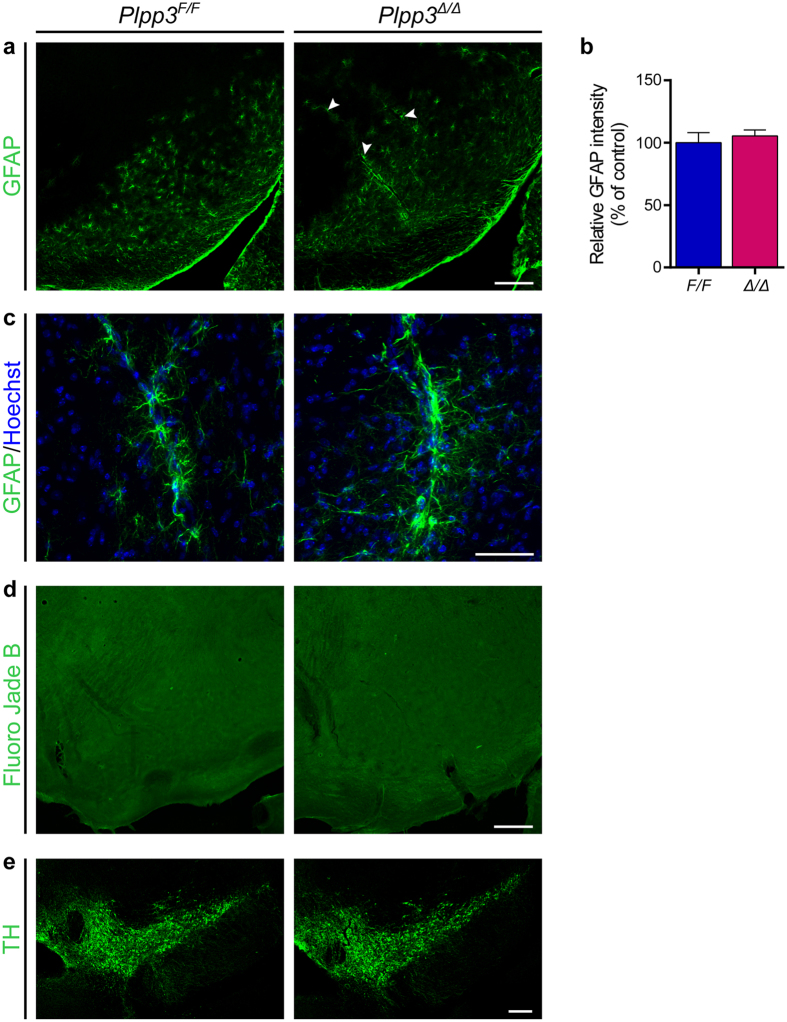
Lack of astrocytic reactivity and Fluoro-Jade^+^ cells in the adult PLPP3-deficient midbrain. **(a)** Immunofluorescent staining for GFAP in the ventral midbrain of 16-week-old *Plpp3*^Δ*/*Δ^ and *Plpp3*^*F/F*^ control mice. Note that GFAP^+^ cells seem more abundant around the blood vessels in the PLPP3 deficient brain (arrowheads). **(b)** Quantitative assessment of GFAP immunoreactivity in the ventral midbrain of *Plpp3*^Δ*/*Δ^ and *Plpp3*^*F/F*^ mice shows no significant differences between genotypes (*p* = 0.3754, *t* test). *Plpp3*^*F/F*^ *n* = 3, *Plpp3*^Δ*/*Δ^ *n* = 3. **(c)** Representative image of perivascular GFAP^+^ astrocytes in the *Plpp3*^Δ*/*Δ^ and *Plpp3*^*F/F*^ ventral midbrain. **(d)** Fluoro-Jade staining on tissue sections across the ventral midbrain of 20-month-old *Plpp3*^Δ*/*Δ^ and *Plpp3*^*F/F*^ mice. No substantial amount of degenerating cells was detected in the SN of either mutant or control mice. **(e)** Antibody staining for TH in the ventral midbrain of 20-month-old *Plpp3*^*F/F*^ and *Plpp3*^Δ*/*Δ^ mice. *Plpp3*^*F/F*^ *n* = 8, *Plpp3*^Δ*/*Δ^ *n* = 11. Scale bars: (**a**,**d**,**e**), 200 μm; (**c**) 50 μm.

Astrocytes become reactive in response to different CNS insults, including pathological conditions and neurodegenerative disorders. Although this cell state —characterised by hypertrophy and upregulation of glial fibrillary acidic protein (GFAP)— is considered a general feature of neuroinflammation, the role of reactive astrocytes in PD is still debatable^31^. To determine whether neural depletion of *Plpp3* may lead to astrocytic reactivity, we analysed GFAP expression in the ventral midbrain of 16-week-old *Plpp3*^Δ*/*Δ^ and control mice by immunofluorescence (Fig. 5a). There were no significant differences in the density of GFAP immunoreactivity between genotypes (Fig. 5b). However, we found that in mice with neural deficiency of PLPP3, there seemed to be accumulation of GFAP^+^ astrocytes around the blood vessels (Fig. 5c).

To confirm the lack of midbrain dopaminergic neurodegeneration in adult PLPP3-deficient mice, we stained tissue sections from 20-month-old animals with Fluoro-Jade to label all possible degenerating cells^32^. No substantial amount of Fluoro-Jade^+^ cells was detected in the ventral midbrain of either control or mutant mice (Fig. 5d). Accordingly, there were no considerable differences in the TH^+^ domains at this age between genotypes (Fig. 5e and Fig. S3). Together with our previous results, these data suggest that CNS-depletion of PLPP3 does not lead to ventral midbrain DA neuron loss, even in aged individuals.

### Non-progressive locomotor impairment in PLPP3-deficient mice

**Figure 6:**
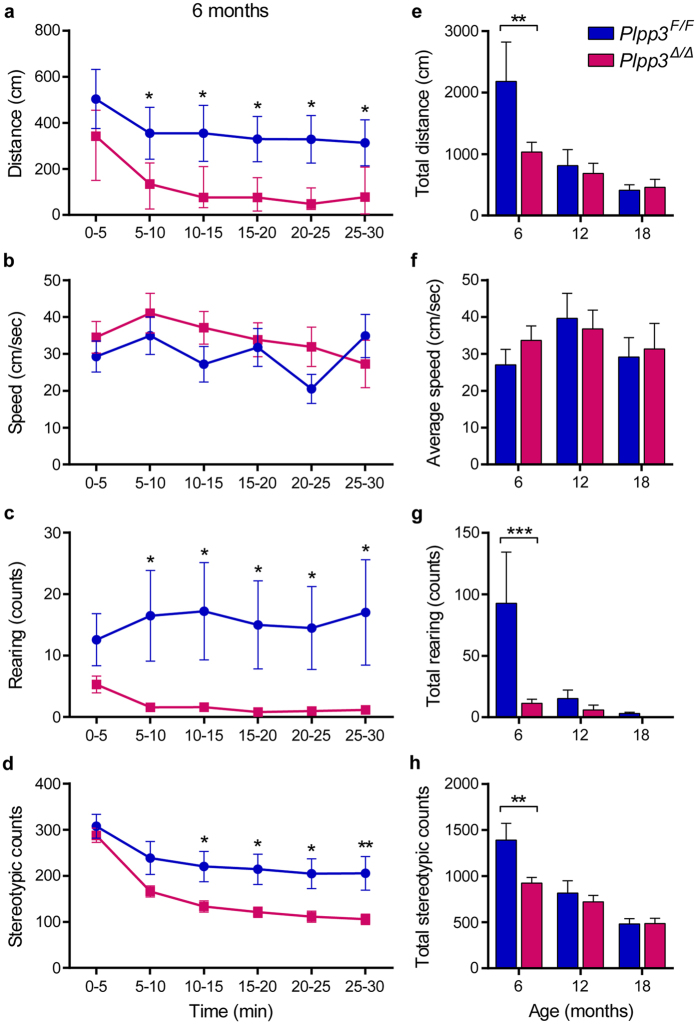
Analysis of spontaneous locomotion in 6- to 18-month-old PLPP3-deficient mice. **(a**–**d)** Locomotor activity was measured for 30 min in mutant (*Plpp3*^Δ*/*Δ^, red squares) and control (*Plpp3*^*F/F*^, blue circles) mice at 6 months of age. Data for each locomotor parameter was analysed using a two-way repeated measures ANOVA. *p < 0.05, **p < 0.005 (*post hoc* tests). **(a)** Distance travelled. Genotype effect (*p* = 0.0244), time effect (*p* < 0.0001), no interaction (*p* = 0.1390). **(b)** Speed. No genotype (*p* = 0.3707) or time (*p* = 0.3684) effects. **(c)** Rearing. Genotype effect (*p* = 0.0093), no time effect (*p* = 0.4927), interaction (*p* = 0.0005). **(d)** Stereotypic movements. Genotype effect (*p* = 0.0042), time effect (*p* < 0.0001), interaction (*p* = 0.0010). **(e**–**h)** Basal locomotor activity is reduced in 6-month-old, but not in 12- and 18-month-old mutant mice. Values represent mean ± standard error of the mean (SEM). Statistically significant differences to age-matched controls are indicated: *p < 0.05, **p < 0.005 (*t* tests). 6 months: distance, speed and stereotypic counts, *Plpp3*^*F/F*^ *n* = 13, *Plpp3*^Δ*/*Δ^ *n* = 28; rearing, *Plpp3*^*F/F*^ *n* = 11, *Plpp3*^Δ*/*Δ^ *n* = 22. 12 months: distance, speed and stereotypic counts, *Plpp3*^*F/F*^ *n* = 14, *Plpp3*^Δ*/*Δ^ *n* = 20; rearing, *Plpp3*^*F/F*^ *n* = 11, *Plpp3*^Δ*/*Δ^ *n* = 17. 18 months: *Plpp3*^*F/F*^ *n* = 10, *Plpp3*^Δ*/*Δ^ *n* = 12.

Using the rotarod and the raised beam tests we previously found that mice with neural deficiency of PLPP3 exhibit a motor dysfunction when compared with age-matched *Plpp3*^*F/F*^ mice. Such deficits were apparent at one month of age and showed no signs of worsening up to 12 months of age^19^. To investigate whether this motor phenotype could be related to parkinsonism, we evaluated the basal activity of 6-, 12- and 18-month-old *Plpp3*^Δ*/*Δ^ mice. At 6 months of age, *Plpp3*^Δ*/*Δ^ animals displayed decreased total locomotion, rearing and stereotypic movements (movement without displacement) when compared with age-matched controls (Fig. 6a,c,d and Fig. S4). These motor deficits became less evident at 12 and 18 months of age, as all animals exhibited an age-related reduction of spontaneous activity (Fig. 6e,g,h and Fig. S5). Yet, a significant impairment of vertical activity was still detected in 12- and 18-month-old mutant mice during distinct phases of the test session (Fig. S5). Collectively, these data indicate that the motor deficit of *Plpp3*^Δ*/*Δ^ mutant mice occurs in the absence of midbrain dopaminergic neurodegeneration and is not progressive, as would be expected for a parkinsonian phenotype.

### Deficits in dopaminergic transmission in *Plpp3*-ablated mice

**Figure 7:**
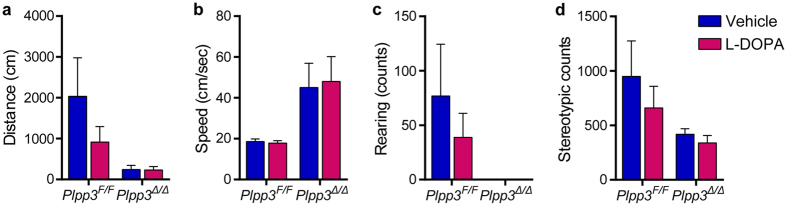
Acute locomotor response of *Plpp3*^Δ*/*Δ^ mice to L-DOPA. **(a**–**d)** The locomotor performance of 7-month-old *Plpp3*^Δ*/*Δ^ and control *Plpp3*^*F/F*^ mice was analysed for 30 min following vehicle injection to obtain baseline activity (blue bars). Animals were then treated with L-DOPA and monitored again (red bars). *Plpp3*^*F/F*^ *n* = 6, *Plpp3*^Δ*/*Δ^ *n* = 8. Values represent mean ± SEM. **(a)** Total distance travelled. Two-way ANOVA: genotype effect (*p* = 0.0095), no treatment effect (*p* = 0.2102) or interaction (*p* = 0.2189). **(b)** Average speed. Two-way ANOVA: genotype effect (*p* = 0.0088), no treatment effect (*p* = 0.9118) or interaction (*p* = 0.8518). **(c)** Vertical activity (rearing counts). Two-way ANOVA: genotype effect (*p* = 0.0164), no treatment effect (*p* = 0.4059) or interaction (*p* = 0.4044). **(d)** Total stereotypic counts. Two-way ANOVA: genotype effect (*p* = 0.0197), no treatment effect (*p* = 0.2903) or interaction (*p* = 0.5449).

Studies with transgenic mouse models of PD suggest that DA neuron dysfunction can occur prior to^33^ or in the absence of neurodegeneration^34^,^35^,^36^. To determine whether the motor symptoms of *Plpp3*^Δ*/*Δ^ mice may result from physiological alterations in the nigrostriatal pathway, we first analysed their acute locomotor response to L-DOPA. We found that L-DOPA administration had no effect on the motor performance of *Plpp3*^Δ*/*Δ^ mice (Fig. 7).

**Figure 8:**
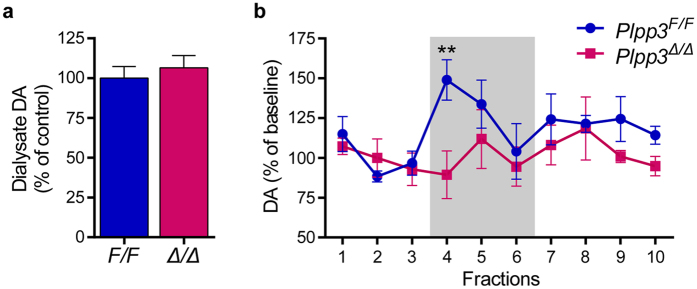
Basal and high potassium-evoked dopamine overflow in *Plpp3*^*F/F*^ and *Plpp3*^*∆/∆*^ mice. *In vivo* microdialysis and capillary electrophoresis were used to assess the levels of basal and stimulus-evoked striatal dopamine overflow in 14- to 17-month old *Plpp3*^*F/F*^ and *Plpp3*^*∆/∆*^ mice. **(a)** There were no differences in the basal levels of striatal extracellular dopamine among genotypes *p* = 0.4861 (*t* test). **(b)** High potassium-evoked overflow of dopamine was reduced in the striatum of PLPP3-deficient mice when compared with age-matched controls. Shaded box indicates perfusion period with high potassium medium. Repeated measures ANOVA: genotype effect (*p* = 0.0462), no dialysate effect (*p* = 0.1817) or interaction (*p* = 0.3355). Fisher’s least significant difference test revealed differences upon high potassium stimulation (Fraction 4) between *Plpp3*^*F/F*^ and *Plpp3*^*∆/∆*^ mice, **p = 0.0013. Values represent mean ± SEM. *Plpp3*^*F/F*^ *n* = 3, *Plpp3*^Δ*/*Δ^ *n* = 3.

Next, we investigated whether neural depletion of PLPP3 may affect dopaminergic transmission. We used *in vivo* microdialysis and capillary electrophoresis to assess basal and high potassium-evoked DA overflow in the striatum of 14 to 17-month-old *Plpp3*^*F/F*^ and *Plpp3*^Δ*/*Δ^ mice. Whereas there were no differences in the basal levels of striatal extracellular DA between genotypes (Fig. 8a), we found that potassium-evoked DA release was significantly reduced in the striatum of PLPP3-deficient mice (Fig. 8b).

## Discussion

Several studies have found an association between the *PARK10* locus and an increased risk for late-onset PD^4^,^5^,^6^,^7^,^37^. However, the biological mechanisms underlying such susceptibility have not been established. Although genetic studies have nominated a set of candidate genes for the *PARK10* locus^7^,^8^,^38^,^39^,^40^, some of these findings have been controversial^41^,^42^,^43^, and until now, further characterisation of these genes in the context of PD had been limited to expression analyses^9^,^44^.

We have performed a functional evaluation of *Plpp3* as a candidate gene for the *PARK10* locus. SAGE profiling of the SN of patients with PD and age-matched controls previously revealed decreased *PLPP3* transcripts in PD tissues^9^. Subsequent work with PD model systems implicated S1P, one of the substrates of PLPP3, in promoting survival of midbrain DA neurons via S1P_1_ activation^16^,^18^. Our studies show that *Plpp3* is expressed in astrocytes throughout the murine nigrostriatal pathway and that its inactivation in CNS progenitor cells causes a severe downregulation of S1P_1_ in the adult ventral midbrain. Yet, we found that PLPP3-deficiency, and the accompanying reduction of S1P_1_ levels, have no significant effect on the number of midbrain DA neurons. Neither do they induce a progressive age-related motor phenotype consistent with PD. This suggests that the changes in *PLPP3* levels detected in patients with PD^9^, and the dysregulation of S1P/S1P_1_ observed in PD murine models^16^, could be a consequence rather than a cause of neuronal loss.

Alternatively, given that PD may result from a complex combination of environmental and genetic factors, the alterations described in this work might increase the vulnerability of midbrain dopaminergic neurons under particular physiological or pathological conditions, so that their effects would only become apparent in specific experimental settings. For instance, dysfunction in the blood-brain barrier (BBB) inevitably leads to inflammatory changes, which in turn contribute to the process of neurodegeneration^45^. In this regard, it is worth noting that although no signs of astrocyte reactivity were detected in the SN of PLPP3-deficient animals, astrocytes seemed to accumulate around blood vessels, being endothelial cells the only cells within the brain that still express PLPP3, and therefore where S1P_1_ was not dysregulated (Fig. S1). This is especially relevant in the light of the recently described role of S1P/S1P_1_ signalling in reinforcing the integrity and function of the BBB by acting on both endothelial cells and astrocytes^46^. We previously showed that targeted ablation of *Plpp3* in endothelial and haematopoietic cells leads to heightened vascular inflammation and permeability^47^. Since in this work we only ablated *Plpp3* in neural tissue, we cannot rule out the possibility that inflammation and increased vascular permeability produced by reduction of PLPP3 levels in endothelial cells could be factors contributing to PD development. In this sense, it would be interesting to explore whether individuals with the *PLPP3* polymorphism associated with increased risk to coronary artery disease^48^, which is susceptible to epigenetic regulation^49^, may also have an increased risk of developing PD.

Despite the lack of any clear signs of neurodegeneration in the ventral midbrain, we found that high potassium-evoked striatal dopamine overflow was significantly decreased in adult mice lacking PLPP3, suggesting alterations in DA release and/or reuptake in the presynaptic nigral terminals. This is particularly interesting since S1P, as well as LPA, another PLPP3 substrate, have been proposed to regulate neurotransmission at different levels^11^,^12^,^13^. In cultured rat hippocampal neurons, for example, S1P has been shown to promote glutamate secretion both in a depolarisation-independent manner and by potentiating depolarisation-evoked release^50^. LPA, on the other hand, has been found to reduce glutamate release by decreasing the number of synaptic vesicles at active zones in rat presynaptic hypoglossal motoneurons, and to reduce GABA signalling by reducing the number of receptors at postsynaptic inhibitory synapses^51^. It would be important to explore whether PLPP3 substrates may exert some effect on DA transport, vesicular release from nigral terminals or on D2 autoreceptors, which modulate the balance between DA release and reuptake.

We previously found that neural inactivation of *Plpp3* leads to abnormalities in cerebellar foliation^19^. Since the cerebellum coordinates sensory and motor functions, it was proposed that the motor phenotype observed in *Plpp3*^Δ*/*Δ^ mice was likely due to defects in cerebellar architecture and/or physiology. However, considering our new findings regarding the deficits in stimulus-evoked striatal DA overflow in PLPP3 mice, at this point we cannot discard a potential contribution of altered dopaminergic transmission to motor impairment. Indeed, it may be possible that the locomotor phenotype detected in this model arises from complex defects in the modulation of neurotransmission in several brain structures, like in the striatum. Further investigation will be required to establish how PLPP3 contributes to this process.

## Methods

### Animals

Studies involving animal use were approved by the Committee of Animal Use and Care of the Instituto de Fisiología Celular and conformed Mexican guidelines (protocols DEA38-14 and LMT01-14). Mice were grouped-housed in an environment with controlled 12 h light/dark cycles and access to food and water *ad libitum*. For *Plpp3* expression analyses, we used CD1 mice heterozygous for a reporter allele of *Plpp3* (*Plpp3*^*lacZ*^)^20^. To deplete PLPP3 in the neural lineage, female 129 mice homozygous for a conditional null allele of *Plpp3* (*Plpp3*^*F/F*^)^25^ were bred to *Plpp3*^*F/F*^ males expressing Cre recombinase under the control of the *Nestin* promoter (*Tg*(*Nes-Cre*)*1Kln*)^26^ in a 129 background. The day of vaginal plug was considered E0.5. All experimental animals were genotyped by PCR analysis of genomic DNA as described^25^. In all analyses, non-excised littermates or age-matched *Plpp3*^*F/F*^ mice were used as controls.

### X-gal staining

Adult mice were anaesthetized by intraperitoneal (i.p.) administration of Avertin (2,2,2-tribromoethanol, 0.375 mg/g body weight, Sigma) and transcardially perfused with 2 mM MgCl_2_/PBS, followed by 25 mL of ice-cold 2% paraformaldehyde (PFA)/2 mM MgCl_2_/PBS. Perfused brains were post-fixed in the same fixative for 2 h on ice and cryoprotected in 20% sucrose/2 mM MgCl_2_/PBS. Fifty μm cryosections were recovered directly onto slides (Superfrost Plus, VWR) and allowed to air-dry overnight. Sections were rinsed twice in 2 mM MgCl_2_/PBS, permeabilised with 0.01% sodium deoxycholate/0.02% IGEPAL/2 mM MgCl_2_/0.1M phosphate buffer (PB) pH 7.3 for 30 min at room temperature (RT) and incubated in staining solution (1 mg/mL X-gal/5 mM K_3_Fe(CN)_6_/5 mM K_4_Fe(CN)_6_/0.01% sodium deoxycholate/0.02% IGEPAL/2 mM MgCl_2_/20 mM Tris pH 7.6/0.1 M PB pH 7.3) for 48 h at 37 °C. Stained sections were rinsed with PBS, post-fixed with 2% PFA, dehydrated in increasing concentrations of ethanol, cleared in xylene and mounted in Permount (Fisher). Transgene-negative littermates were used as controls for background staining.

### Whole-mount immunohistochemistry

Methods for whole-mount antibody staining were adapted from previously established protocols^52^. Dissected brains from E14.5 embryos were fixed in phosphate buffered 4% PFA overnight at 4 °C, rinsed in PBS and sequentially dehydrated in methanol. After 6 h incubation in methanol/DMSO/H_2_O_2_ (4:1:1) at RT, tissues were washed twice with 100% methanol, rehydrated in descending concentrations of methanol/PBS and rinsed twice with PBS. Samples were incubated in an ice-cold blocking solution containing 2% skim milk and 0.5% Triton X-100 in PBS (PBSTM) for 2.5 h on a rocker. Primary polyclonal rabbit anti-TH antibody (1:250, Millipore AB152) was applied in PBSTM overnight at 4 °C with gentle rocking. Following five 1 h washes with PBSTM and constant rocking, tissues were incubated with HRP-conjugated anti-rabbit secondary antibody (1:500, Santa Cruz sc-2030) in PBSTM overnight at 4 °C on a rocker. After five 1 h washes with PBSTM and two PBS rinses, tissues were incubated with 3-3′-diaminobenzidine/NiCl_2_ (DAB Kit, Vector) for 30 min in the dark, before adding H_2_O_2_. Stained brains were rinsed with dH_2_O, post-fixed in 4% PFA, rinsed with PBS, dehydrated in ascending methanol concentrations, cleared in benzyl alcohol/benzyl benzoate (1:1) and photographed under a stereomicroscope (Nikon SMZ800).

### Immunofluorescence and Fluoro-Jade staining

Dissected brains from E14.5 embryos were fixed for 2 h in 4% PFA at 4 °C, cryoprotected in 30% sucrose/PBS and embedded in OCT. Coronal 20 μm cryosections were collected directly onto slides. Adult mice were anaesthetized with Avertin and perfused with PBS, followed by ice-cold 4% PFA. Perfused brains were post-fixed in 4% PFA overnight at 4 °C and cryoprotected in 30% sucrose/PBS. Forty μm sections were cut on a cryostat and maintained free-floating. Sections were washed twice with PBS and incubated with blocking solution at RT for 1–2 h. Embryonic tissues were blocked in 1% bovine serum albumin (BSA)/1% serum/0.1% Triton-X 100/PBS. For adult tissues, a solution containing 5% serum/0.2% Triton-X 100/PBS was used. Primary antibodies were applied in blocking solution for 36–48 h at 4 °C. Following three washes with PBS, sections were incubated with AlexaFluor-conjugated secondary antibodies (1:500, Life Technologies) in blocking solution O/N at 4 °C. Samples were rinsed thrice with PBS and nuclei were counterstained with Hoechst-33342, prior to mounting in Vectashield (Vector) or Fluoromount-G (SouthernBiotech). For PLPP3 staining, sections were adhered onto slides, air-dried overnight and treated with antigen unmasking solution (Vector) for 6.5 min in a pressure cooker before blocking. Primary antibodies were: rabbit anti-EDG1/S1P_1_ (H-60) (1:200, Santa Cruz sc-25489), rabbit anti-GFAP (1:500, DAKO Z0334), rat anti-GFAP (1:500, Invitrogen 13-0300), mouse anti-GLAST (1:300, Millipore MABN794), rabbit anti-TH (1:500, Millipore AB152), sheep anti-TH (1:500, Pel-Freez P60101), and a rabbit anti-PLPP3 (1:300, Sigma, custom-made) generated against a peptide antigen spanning the residues 2–7 of human PLPP3^22^.

Fluoro-Jade B (FJB) staining was performed as previously described^53^. One drop of a 1% NaOH solution diluted in 80% ethanol was added to brain sections adhered onto slides and replaced 2 min later by 70% ethanol. Sections were incubated in 0.06% potassium permanganate for 10 min, washed, and incubated 20 min with 0.0004% FJB solution in 0.1% acetic acid. Sections were washed, dried at 50 °C, cleared in xylene for 5 min and mounted in Permount.

Confocal images were acquired using a FV10i (Olympus), a FV1000 (Olympus) or a LSM710 NLO microscope (Zeiss), and processed using ImageJ (NIH, USA) and Photoshop (Adobe). For mosaic assembly, we used ImageJ Stitching plugin^54^.

### Cell counts and measurement of striatal TH^+^ fibre and midbrain GFAP^+^ astrocyte densities

For 16-week-old animals, the number of TH^+^ somata in the entire extent of the SN-VTA was counted by unbiased stereology according to the optical fractionator principle, using an Olympus BX51WI spinning disc confocal microscope and the StereoInvestigator software (MicroBrightField Biosciences). Number of TH^+^ somata in *Plpp3*^Δ*/*Δ^ samples is given as a percentage of the controls. Density of dorsolateral striatal TH^+^ fibres was measured at two different coronal levels in each hemisphere using ImageJ. Mean grey values obtained from the striatum were normalised using corresponding measurements from the adjacent cortex. Assessment of GFAP^+^ immunofluorescence intensity in the SN was performed using ImageJ, as previously reported^55^. Measurements were done on confocal images of each hemisphere acquired at two different coronal levels, and corrected for not specific background staining. Average values for each mouse were used for statistical analyses.

### Immunoblotting

Adult mice (10- to 12-month-old) were euthanized by cervical dislocation, brains were removed and placed in ice-cold Krebs buffer solution. Cerebella and ventral midbrain were dissected and lysed in P40 extraction buffer consisting of 50 mM Tris-HCl pH 8.0/150 mM NaCl/1% IGEPAL/1 mM Na_2_VO_3_/25 mM NaF/1X cOmplete EDTA-free protease inhibitor cocktail (Roche Molecular Diagnostics). The samples were homogenised, centrifuged at 13000 × *g* for 3 min and cleared supernatants were collected. Protein concentration was determined using DC Protein Assay Reagents (Bio-Rad) and BSA as the standard. Forty micrograms of total protein were separated by SDS-PAGE and electroblotted onto polyvinylidene fluoride (PVDF) membranes (Millipore). Membranes were blocked in 6% (w/v) milk/TBS-Tween (TBS-T). Only for TH antibody, 5% (w/v) BSA in TBS-T was used as blocking solution. Primary antibodies were applied in blocking solution overnight at 4 °C. Following three washes with TBS-T, species-specific HRP-conjugated secondary antibodies (Santa Cruz) were applied at 1:5000 dilution for 1h at RT. Peroxidase activity was visualized using ECL Western Blotting Analysis System (Amersham, GE Healthcare) or Immobilon Western Chemiluminescent HRP substrate (Millipore). Primary antibodies were: rabbit anti-PLPP3 (1:2000, Sigma, custom-made), rabbit anti-EDG1/S1P_1_ (H-60) (1:3000, Santa Cruz), rabbit anti-TH (1:2000, Millipore) and mouse anti-GAPDH (1:3000, Millipore MAB374). In agreement with previous reports, the antibody against PLPP3 detected a faster migrating band corresponding to the non-glycosylated form of the enzyme, as well as slower migrating bands representing glycosylated forms^22^,^56^. Densitometric analyses were performed using ImageJ.

### *In vivo* microdialysis and capillary electrophoresis

Microdialysis experiments were performed following the methodology reported by Camacho and colleagues^57^, adapted to the mouse. Adult mice were anaesthetized with 1.5% isoflurane and maintained under low (0.8%) anaesthesia throughout the procedure. Animals were fixed on a stereotaxic frame for unilateral implantation of a 1 mm long and 0.5 mm diameter microdialysis probe (CMA 12, CMA Microdialysis) in the dorsal striatum at the coordinates: AP 0.6 mm, ML 1.8 mm and DV 3.2 mm, relative to bregma and the skull surface and according to Paxinos and Franklin^58^. The probe was connected to a micro infusion pump (Harvard Apparatus 22), keeping constant flow of basal Krebs-Ringer bicarbonate-buffered (KRB) medium containing 118 mM NaCl/4.7 mM KCl/1.2 mM MgSO_4_/1.2 mM KH_2_PO_4_/2.5 mM CaCl_2_/19 mM NaHCO_3_/3.3 mM glucose, at a rate of 0.25 μL/min. After 60 min perfusion with KRB (stabilisation time), 4 μL fractions were continuously collected every 16 min. Following collection of three baseline dialysate fractions, perfusate was changed to a high-potassium Ringer’s solution (82 mM NaCl/110 mM KCl/1.2 mM MgSO_4_/1.2 mM KH_2_PO_4_/2.5 mM CaCl_2_/19 mM NaHCO_3_/3.3 mM glucose) and three fractions were collected in these conditions. Solution was switched back to KRB medium before collecting the last four fractions. Dialysates were collected in tubes containing 1 μL of antioxidant mixture (0.25 mM ascorbic acid/0.27 mM Na_2_EDTA/0.1 M acetic acid) and immediately frozen at −80 °C.

Dialysate DA levels were determined by micellar capillary electrophoresis^59^. For derivatisation, 6 μL of 2.5 mg/mL 3-(2-furoyl)quinoline-2-carboxaldehyde (Molecular Probes, Eugene, OR, USA) were added to each striatal microdialysate sample in the presence of 2 μL of KCN 25 mM/10 mM borate buffer pH 9.2 and 1 μL of 0.075 mM O-methyl-L-threonine as internal standard. The derivatisation reaction was carried out in the dark at 65 °C for 15 min. Separation and analysis were conducted on a capillary electrophoresis system (Beckman-Coulter P/ACE MDQ Glycoprotein System, Beckman Coulter, Brea, CA, USA) with laser induced fluorescence detection. An argon ion laser was used to excite the 3-(2-furoyl)quinoline-2-carboxaldehyde-labeled analytes with light at 488 nm. The separation of compounds was based on a micellar electrokinetic chromatography buffer system that included 35 mM borates/25 mM sodium dodecyl sulphate/13% (vol/vol) methanol HPLC grade, pH 9.6. The dialysate samples were injected hydrodynamically at 0.5 psi for 5 seconds in a 75 μm i.d. capillary (Beckman Coulter); the separation was performed at 25 kV. After each sample separation, the capillary was flushed with 0.1 M NaOH, H_2_O and running buffer. In order to identify dopamine, migration electropherograms were matched with a spiked sample. Samples were corrected by relating the area under the curve of the unknown sample with the area under the curve of the internal standard. Data were analysed using Karat System Gold (Beckman Coulter). Basal dialysates DA levels are given as percentage of controls. Results for all the perfusion period are expressed as percentage of baseline concentration (analyte concentration × 100/mean of the three first fractions).

### Locomotor tests

Spontaneous locomotor activity was evaluated in an automatic activity monitor (Med Associates Inc.) during the light phase of the light/dark cycle. Mice were individually placed in the centre of a 43 × 43 cm open-field arena and distance travelled, stereotypic movements, rearing and time spent in the centre of the arena (45% of total surface) were measured in sequential 5 min intervals during a 30 min test period. The arena was thoroughly cleaned with 10% isopropanol after each test session. For the analysis of motor phenotype progression, each animal was tested for 3 consecutive days at 6, 12 and 18 months of age. Mean values of each three-day trial were used for statistical analyses. For assessment of time spent in the centre of the arena, only values of the first test session at 6 months of age were used.

To evaluate response to L-DOPA, 7-old-month mice were tested for 4 consecutive days. On the first 2 days, each animal was given a saline injection and their activity was subsequently monitored for 30 min as indicated above. On the next 2 days, mice were treated with L-DOPA combined with the peripheral DOPA-decarboxylase inhibitor benserazide (20 mg/kg; Sigma) prior to locomotor evaluation. All locomotor tests were carried out with male mice.

### Statistical Analyses

For TH^+^ neuron counts, densitometric measurements of TH^+^ striatal fibres and GFAP immunoreactivity, immunoblot densitometry and basal extracellular dopamine levels within striatum, means comparisons were done using two-tailed *t* tests. For spontaneous locomotor activity, data for each locomotor parameter were analysed within each age group using a repeated measures (time intervals) analysis of variance (ANOVA), followed by Bonferroni *post hoc* tests (when there was a significant time/genotype interaction) or the Fisher’s least significant difference test (when there was no time/genotype interaction). Two-tailed *t* tests were used to evaluate differences in average total distance, mean speed, total rearing and total stereotypic movements between genotypes within a given age group. The effects of L-DOPA treatment on each locomotor parameter in both control and *Plpp3*^Δ*/*Δ^ mice were assessed using a two-way ANOVA, with genotype and treatment as the two factors. Genotype effects on changes in striatal dopamine overflow induced by high potassium were analysed using a repeated measures (dialysates) ANOVA, followed by the Fisher’s least significant difference test. All analyses were performed with GraphPad Prism (GraphPad Software). The level of significance was *p* ≤ 0.05.

## Additional Information

**How to cite this article**: Gómez-López, S. *et al*. Neural ablation of the *PARK10* candidate *Plpp3* leads to dopaminergic transmission deficits without neurodegeneration. *Sci. Rep*. **6**, 24028; doi: 10.1038/srep24028 (2016).

## Supplementary Material

Supplementary Information
