# Supplementary material for: Neural ablation of the PARK10 candidate Plpp3 leads to dopaminergic transmission deficits without neurodegeneration
Source: Sci Rep. 2016 Apr 11;6:24028. doi: 10.1038/srep24028 (PMC4827058; doi:10.1038/srep24028)
Supplement: Supplementary Information [file srep24028-s1.pdf]

Supplementary Information

Neural ablation of the *PARK10* candidate *Plpp3* leads to dopaminergic transmission deficits without neurodegeneration

Sandra Gómez-López, Ana Valeria Martínez-Silva, Teresa Montiel, Daniel Osorio-Gómez, Federico Bermúdez-Rattoni, Lourdes Massieu and Diana Escalante-Alcalde

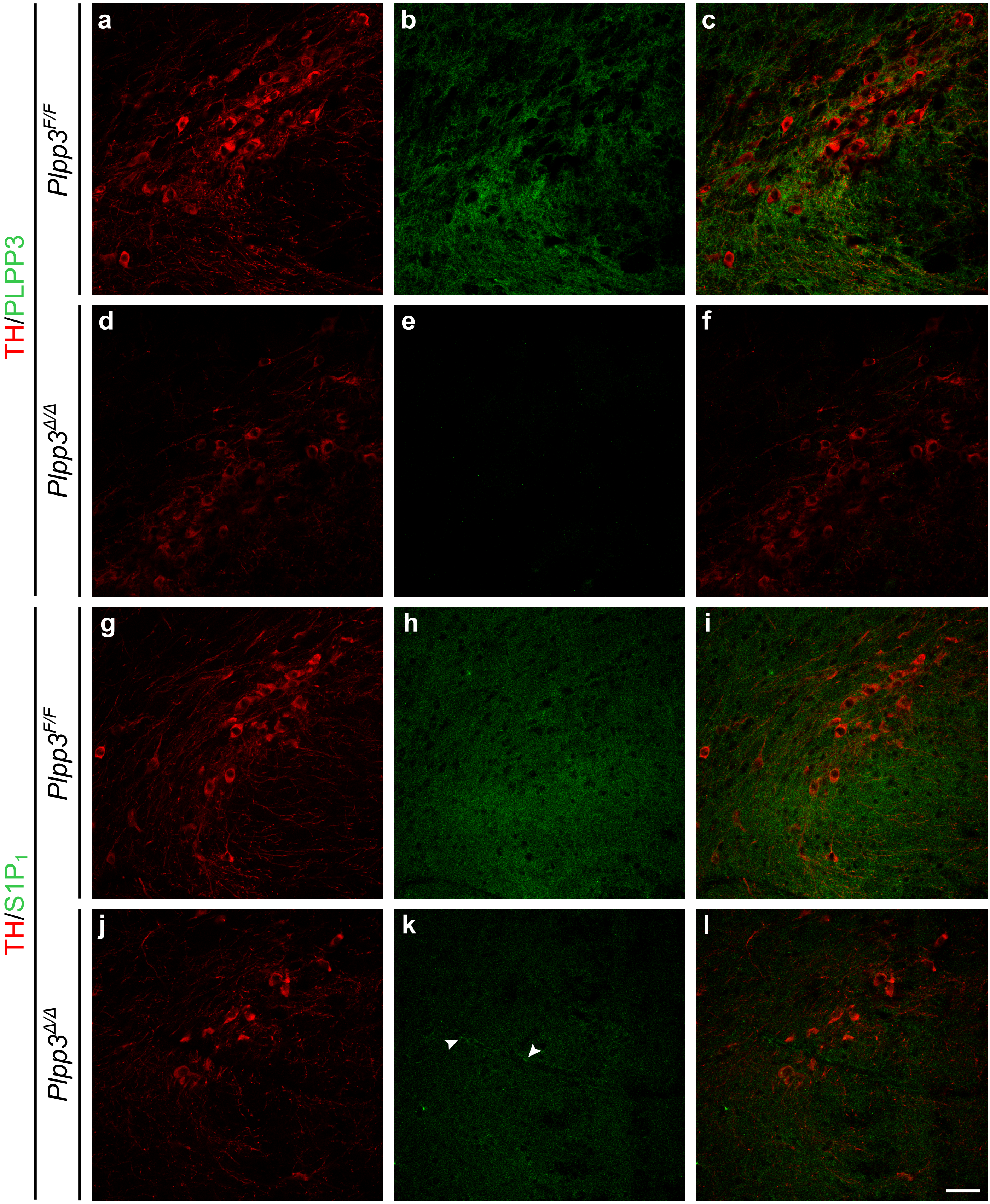

Figure S1. PLPP3-deficiency in the adult ventral midbrain. Double immunofluorescence for TH and PLPP3 on coronal sections from 16-week-old **(a-c)** *Plpp3*$^{F/F}$ and **(d-f)** *Plpp3*$^{Δ/Δ}$ mice showing loss of PLPP3 immunoreactivity in the *Plpp3*$^{Δ/Δ}$ midbrain. Immunostaining for TH and $S1P_1$ in the **(g-i)** *Plpp3*$^{F/F}$ and **(j-l)** *Plpp3*$^{Δ/Δ}$ ventral midbrain. Note that in the *Plpp3*$^{Δ/Δ}$ tissue $S1P_1$ expression is reduced in neural but not in endothelial cells (arrowheads in k). Scale bar, 50 μm.

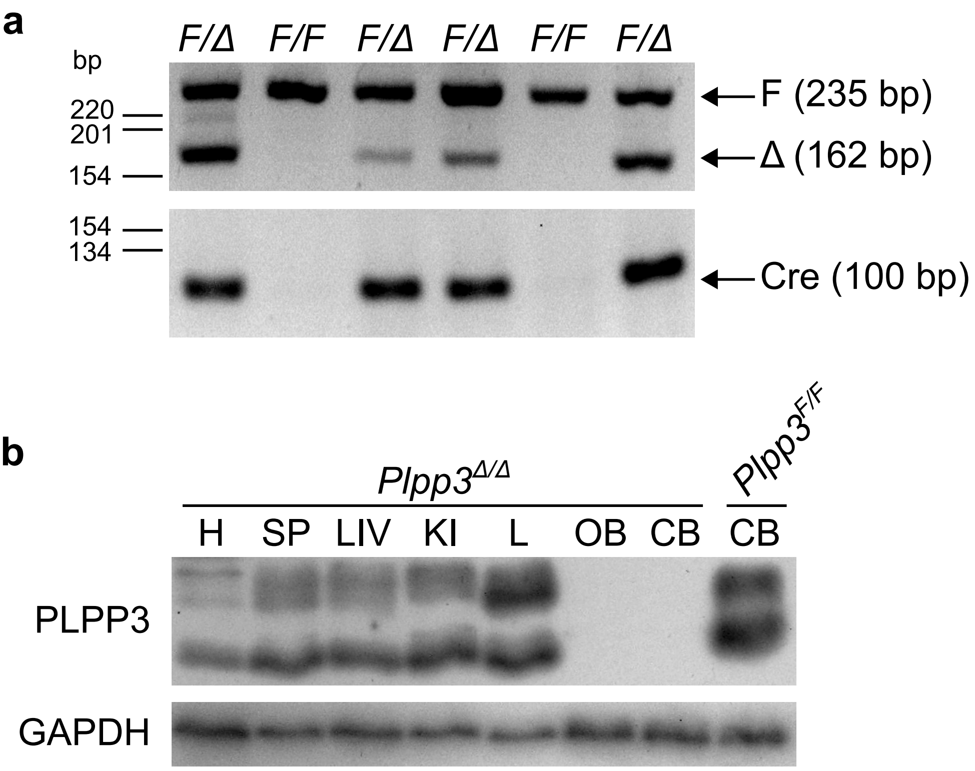

Figure S2. Conditional inactivation of *Plpp3* in the neural lineage. **(a)** Genotype analysis of mice obtained from *Plpp3*$^{F/F}$ × *Plpp3*$^{F/F}$;*Nestin::Cre* crosses. PCR product sizes are indicated on the right. Given that genomic DNA used for genotyping is extracted from tail biopsies that contain both neural and non-neural tissue, the PCR product for the floxed allele (*F*) is still amplified in mice expressing *Nestin::Cre*. **(b)** Western blot analysis for PLPP3 showing central nervous system-specific depletion of the enzyme in *Plpp3*$^{F/F}$;*Nestin::Cre* (*Plpp3*$^{\Delta/\Delta}$) mice. No excision is observed in the *Plpp3*$^{F/F}$ cerebellum (CB) in the absence of Cre. H, heart; SP, spleen; LIV, liver; KI, kidney; L, lung; OB, olfactory bulb.

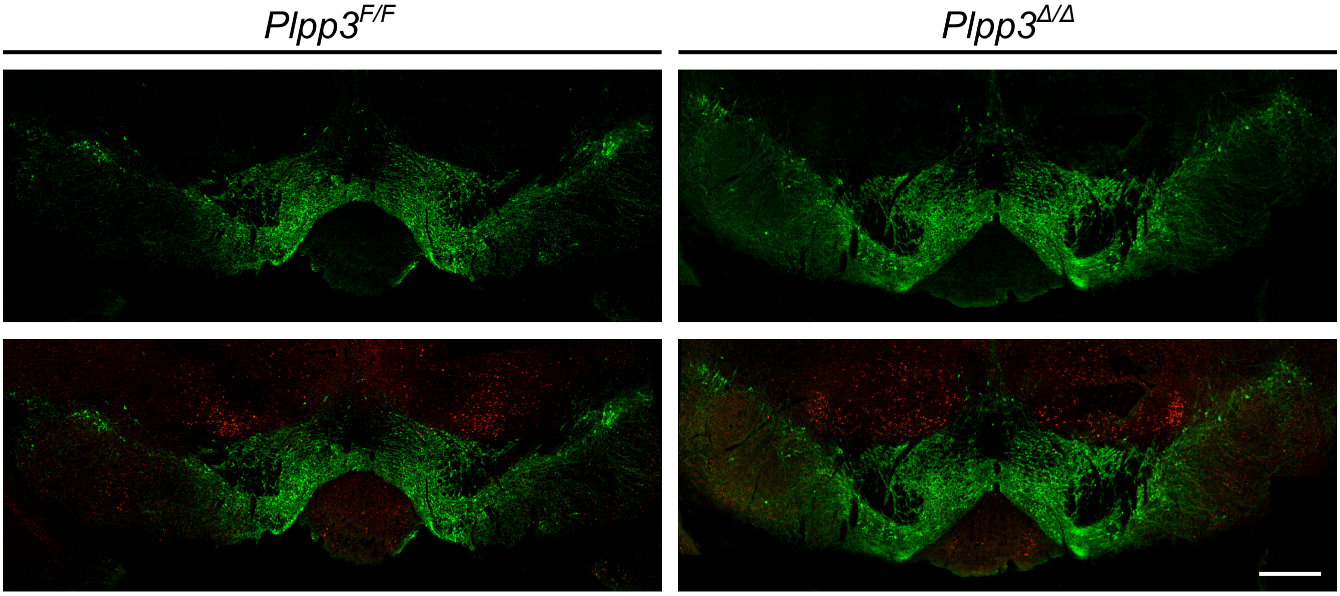

Figure S3. Antibody staining for TH in the ventral midbrain of 20-month-old mice. TH immunofluorescence (green) on coronal sections from *Plpp3*$^{F/F}$ and *Plpp3*$^{\Delta/\Delta}$ mice shows similar distribution and abundance of TH^{+} signal in the SN and VTA. The typical red autofluorescence (under 540 nm excitation light) of the aging pigment lipofuscin is also similar between genotypes and confirms the advanced aging of the analysed individuals. Scale bar 400 μm.

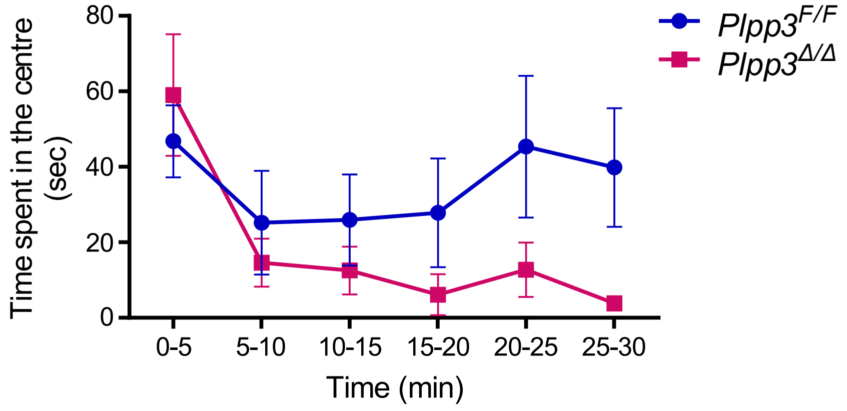

Figure S4. Time spent in the centre of an open-field arena. 6-month-old *Plpp3*$^{\Delta/\Delta}$ and *Plpp3*$^{F/F}$ mice were placed in an open-field arena and the time they spent in the central area encompassing 45% of the total surface was measured during a 30 min test period. Values represent mean ± SEM. Two-way repeated measures ANOVA revealed a time effect (p<0.0001) and interaction (p=0.0311), but no genotype effect (p=0.1630). *Plpp3*$^{F/F}$ n=11, *Plpp3*$^{\Delta/\Delta}$ n=17.

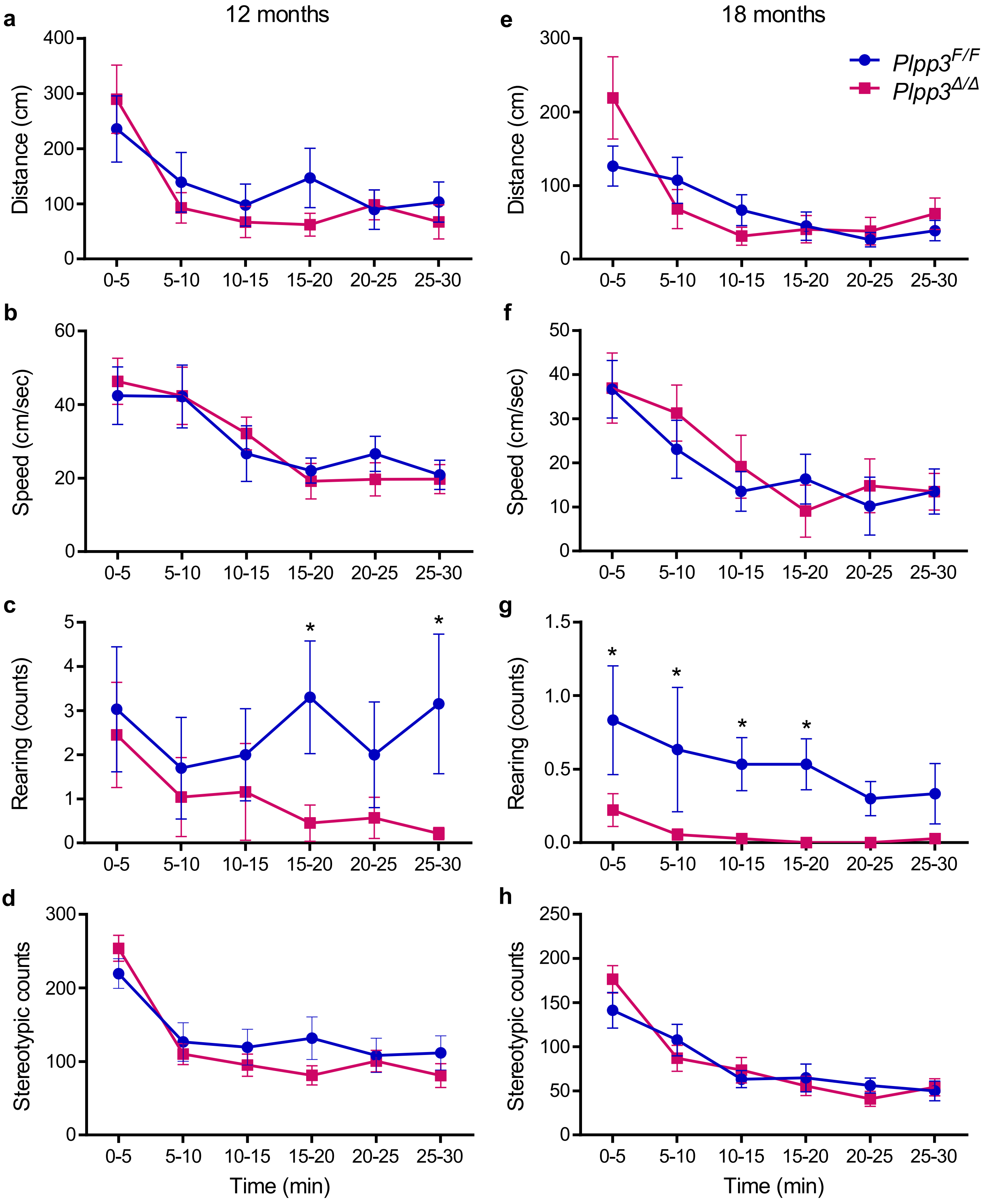

Figure S5. Analysis of spontaneous activity in 12- and 18-month-old PLPP3-deficient mice. Locomotor activity was measured for 30 min in mutant (*Plpp3*$^{Δ/Δ}$, red squares) and control (*Plpp3*$^{F/F}$, blue circles) mice at **(a-d)** 12 and **(e-h)** 18 months of age. 12 months: distance, speed and stereotypic counts, *Plpp3*$^{F/F}$ *n*=14, *Plpp3*$^{Δ/Δ}$ *n*=20; rearing, *Plpp3*$^{F/F}$ *n*=11, *Plpp3*$^{Δ/Δ}$ *n*=17. 18 months: *Plpp3*$^{F/F}$ *n*=10, *Plpp3*$^{Δ/Δ}$ *n*=12. Values represent mean ± SEM. *p<0.05 (*post hoc* tests).
